# Effects of High-Concentrate Diets on Growth Performance, Serum Biochemical Indexes, and Rumen Microbiota in House-Fed Yaks

**DOI:** 10.3390/ani14243594

**Published:** 2024-12-12

**Authors:** Ben Zhang, Xingdong Wang, Ziqiang Ding, Yandong Kang, Shaoke Guo, Mengli Cao, Liyan Hu, Lin Xiong, Jie Pei, Xian Guo

**Affiliations:** 1 Key Laboratory of Yak Breeding in Gansu Province, Lanzhou Institute of Husbandry and Pharmaceutical Sciences, Chinese Academy of Agricultural Sciences, Lanzhou 730050, China; zhangbencaas@163.com (B.Z.); dingziqiang1997@163.com (Z.D.); kangyandong0901@163.com (Y.K.); gsk1125@163.com (S.G.); caomengliaaa@163.com (M.C.); huliyan2020@163.com (L.H.); xionglin@caas.cn (L.X.); 2 Key Laboratory of Animal Genetics and Breeding on Tibetan Plateau, Ministry of Agriculture and Rural Affairs, Lanzhou 730050, China; 3 College of Life Science and Technology, Inner Mongolia Normal University, Hohhot 010022, China; wxd17339929758@163.com

**Keywords:** yak, high concentrate, growth performance, serum indexes, rumen microbiota

## Abstract

The aim of this study is to determine the effects of a high-concentrate diet on growth performance, serum biochemical indexes, and rumen microbiota in house-fed yaks. Sixteen male yaks (body weight, 151.73 ± 14.11 kg; 18 months) were randomly allocated into two dietary treatments: a group with a low level of concentrate (*n* = 8, LC, concentrate–forage = 40:60) and a group with a high level of concentrate (*n* = 8, HC, concentrate–forage = 60:40). We found that compared with the LC group, the average daily feed intake (ADFI), the average daily gain (ADG), and the serum albumin (ALB) concentration in the HC group were significantly increased (*p* < 0.05). The rumen bacterial compositions also differed significantly between the groups, as indicated by principal coordinate analysis (*p* < 0.05). *Firmicutes* and *Bacteroidota* were the main dominant phyla of rumen bacteria in yaks. Compared with the LC group, the relative abundance of *Firmicutes* in the HC group was significantly increased (*p* < 0.05) and the relative abundance of *Bacteroidota* was significantly decreased (*p* < 0.05). At the genus level, *Rikenellaceae_RC9_gut_group*, *Succiniclasticum*, *Prevotella*, *Christensenellaceae_R-7_group*, and *NK4A214_group* had the highest relative abundance. The relative abundance of *Christensenellaceae_R-7_group* in the HC group was significantly higher than that in the LC group (*p* < 0.05). The PICRUSt 2 results showed a significant enrichment in glycosaminoglycan degradation, apoptosis, and ECM–receptor interaction in HC relative to LC (*p* < 0.05). In conclusion, high-concentrate diets can enhance growth performance and alter the compositions and functions of ruminal bacterial communities in yaks.

## Simple Summary

In order to increase the economic benefits of breeding, local people often use a high-concentrate diet to feed yaks. The appropriate concentrate ratio can improve rumen fermentation and production performance. However, long-term feeding with a high-concentrate diet can induce a series of nutritional metabolic diseases. At present, there are limited reports on the effects of a high-concentrate diet on growth performance, serum biochemistry, and rumen flora in yaks. This study suggested that high-concentrate diets raised the average daily feed intake (ADFI), average daily gain (ADG), and albumin (ALB) concentration. The relative abundance of *Firmicutes*, *Christensenellaceae_R-7_group*, and *NK4A214_group* was greater in the HC group, while the levels of glycosaminoglycan degradation, apoptosis, and ECM–receptor interactions were greater in the LC group. These results provide a basis for the utilization of diets containing high concentrate levels in yak fattening and production.

## 1. Introduction

The yak (*Bos grunniens*), endemic to the Qinghai–Tibet Plateau (QTP), exhibits remarkable adaptability to challenging conditions, including high altitude, extreme cold, low air pressure, and low oxygen content [1,2]. Globally, the yak population is about 17.5 million, of which 94.4% can be found in China, where they are a cornerstone of the local economy and livelihoods by supplying livestock products such as meat, wool, milk, and dung to local herders [3,4]. Influenced by the special geographical environment of the QTP and the traditional concept of local herders, the yak feeding model is still dominated by grazing [5]. The division of the four seasons on the plateau is not obvious, and there are only two seasons in a year. Forage grass is usually lacking between October and April, while in the warmer weather from May to September, there are abundant forage grass resources [6]. In particular, in the long cold season, grazing yaks often show a low calving rate and growth rate and other adverse phenomena [3,7]. At the same time, due to the increasing number of yaks, the grassland has been degraded to different degrees [8]. Therefore, in recent years, based on ecological protection, market demand and breeding benefits, and other factors, yak husbandry has evolved from traditional grazing to large-scale house-feeding and fattening [9,10].

The concentrate-to-forage ratio is the basis for the scientific feeding of ruminants [11], which directly affects the nutritional intake, digestion and absorption, and health status of ruminants. However, in production practice, in order to promote the intake of energy, protein, minerals, and vitamins in the diet of ruminants and improve economic benefits, farmers generally adopt high-concentrate feeding [12,13]. While the proper proportion of concentrate can increase fat deposition and optimize feed utilization efficiency in ruminants [14,15], prolonged feeding with high-concentrate diets can cause rumen metabolic disorders, rumen acidosis, and other metabolic diseases, which seriously damages the feed conversion rate, gastrointestinal functions, and overall animal health [16,17]. Consequently, identifying the optimal level of concentrate is important in ensuring yak growth and well-being under house-feeding conditions.

The current research on the influence of high-concentrate diets has primarily focused on sheep [9], goats [18], and dairy cows [19], while there are few studies on house-fed yaks. It was hypothesized that a diet with 60% concentrate would promote growth performance, improve blood biochemical parameters, and alter the microflora composition. Therefore, this study explored the effects of a diet containing 60% concentrate on the growth, serum biochemical indices, and rumen microbiota of yaks, providing a basis for optimizing diet formulations for yaks under the condition of house feeding.

## 2. Materials and Methods

The animal protocol followed the guidelines of the Ministry of Agriculture of the People’s Republic of China and the Chinese Animal Protection Commission. This study also received approval from the Animal Protection and Utilization Committee of the Lanzhou Institute of Husbandry and Pharmaceutical Sciences, Chinese Academy of Agricultural Sciences (License No. SYXK-2014-0002).

### 2.1. Animals and Experimental Design

This experiment, performed between October and December 2023 at the professional cooperative of yak breeding in Kecai Town, Xiahe County, Gannan Tibetan Autonomous Prefecture, Gansu Province (34.64° N, 102.23° E, altitude 3260 m), involved sixteen 18-month-old healthy male yaks with a body weight of 151.73 ± 14.11 kg and a normal appetite. The yaks were randomly divided into two groups with eight per group and were given a total mixed ration (TMR), with one group fed with a low level of concentrate (LC, concentrate–forage = 40:60) and the other group fed with a high level of concentrate (HC, concentrate–forage = 60:40). The pre-experimental period was 10 days, and the formal experimental period was 90 days. During the experiment, the natural grassland in the area was in a period of hay grass, and the vegetation cover was sparse.

### 2.2. Diets and Feeding Management

The diet design was based on the Chinese Beef Cattle Feeding Standard (NY/T815-2004). Both groups had diets with nearly identical energy and protein content. The dietary composition and nutrient levels are shown in Table 1. The yaks were fed daily at 08:30 and 17:30, and prior to each feeding, the feed intake was recorded to ensure the availability of surplus feed for the following day. Throughout the experiment, food and water were freely consumed.

### 2.3. Sampling and Measurement

The formal experiment was divided into different stages, 0–30 d, 31–60 d, 61–90 d, and the whole period, 0–90 d. In each stage of the experiment, the average daily feed intake (ADFI) of each yak was assessed from daily measurements of the actual TMR feed and residual feed. Before morning feeding on days 0, 30, 60, and 90 of the formal experiment, the animals were weighed using a weighbridge, and the average daily gains (ADGs) of the 0–30-, 31–60-, 61–90-, and 0–90-day periods were calculated, respectively. ADFI and ADG were calculated as follows.

$$\mathrm{ADFI}=\frac{\mathrm{feed}\;\mathrm{amount}-\mathrm{residue}\;\mathrm{amount}}{\mathrm{experimental}\;\mathrm{days}}$$

$$\mathrm{ADG}=\frac{\mathrm{final}\;\mathrm{weight}-\mathrm{initial}\;\mathrm{weight}}{\mathrm{experimental}\;\mathrm{days}}$$

On days 30, 60, and 90 of the formal experiment, all the experimental yaks were selected for fasting jugular vein blood collection in the morning. The blood obtained from each yak (5 mL) was subjected to a 15 min centrifugation at 4500 r/min, with the transfer of the sera to fresh 1.5 mL centrifuge tubes, followed by freezing at −20 °C and transport to the laboratory. When required, these isolated serum samples were thawed on ice to determine the concentrations of glucose (GLU), creatinine (CREA), globulin (GLB), albumin (ALB), total protein (TP), lactate dehydrogenase (LDH), alkaline phosphatase (ALP), alanine aminotransferase (ALT), and aspartate aminotransferase (AST) in serum using a Mindray BS-420 automatic biochemical analyzer (Shenzhen Mindray Biomedical Electronics Co., Ltd., Shenzhen, China).

At the end of the experiment, all yaks were subjected to one day of fasting before the collection of ruminal fluid (50 mL) using an oral gastric tube, with the fluid then passed immediately through 4 layers of sterile gauze before storage at −80 °C for analysis.

### 2.4. DNA Extraction, PCR, and Sequencing

Total microbial DNA was extracted from ruminal fluid with a HiPure Stool DNA extraction Kit (Magen, Guangzhou, China), in accordance with the directions provided. The DNA was evaluated by electrophoresis to evaluate DNA integrity; the NanoDrop 2000 microspectrophotometer (Thermo Scientific, Wilmington, DE, USA) was used to determine the DNA concentration and purity. The V3-V4 variable regions of the 16S rDNA genes were amplified. The primers were 341 F (5′-CCTACGGGNGGCWGCAG-3′) and 806 R (5′-GGACTACHVGGGTATCTAAT-3′), and the conditions were 5 min predenaturation at 95 °C, 30 cycles of 1 min denaturation at 95 °C, 1 min annealing at 60 °C, and 1 min extension at 72 °C, with 7 min extension at 72 °C. In this case, each 50 µL mixture consisted of 0.2 µL Q5 High-Fidelity DNA Polymerase, 1.5 µL upstream and downstream primers (10 µM), 1.5 µL dNTP (2.5 mmol/L), 10 µL 5 × Q5 High GC Enhancer, 10 µL 5 × Q5 Reaction Buffer and 50 ng template DNA, supplemented with H_2_O to 50 µL. After quality assessment on 2% agarose gels, the products were purified and quantified using AMPure XP Beads (Beckman, Brea, CA, USA) and Qubit 3.0, respectively. An Illumina DNA Prep Kit (Illumina, CA, USA) was then used to construct sequencing libraries, and after assessing their quality with an ABI StepOnePlus Real-Time PCR System (Life Technologies, Foster City, CA, USA), sequencing was performed in PE250 mode on Novaseq 6000. The raw data have been uploaded to the NCBI Sequence Read Archive (accession no. PRJNA1173922).

### 2.5. Sequencing Data Processing and Analysis

The raw Illumina sequencing data were filtered using FASTP [20] (V0.18.0) before using FLASH [21] (V1.2.11), with a maximum mismatch rate of 2% and a minimum overlap of 10 bp to combine the clean reads into tags. Those of low quality were then filtered using the filtering conditions described in reference [22] to produce the high-quality clean tags. Using the UPARSE [23] (V9.2.64) process, clean tags were grouped into operational taxonomic units (OTUs; minimum 97% similarity). Chimeras were identified and filtered with the UCHIME algorithm [24] before OTU analysis. For each OTU, the most abundant tag was chosen as a representative [25]. To obtain taxonomic information for each species, the RDP classifier (V2.11) was used for the classification and annotation of the representative sequences using the Silva database [26], with a 70% threshold. Alpha diversity indices were obtained by using the QIIME software (V1.9.1), including Shannon, Chao1, and ACE. Weighted UniFrac distances were also utilized for the calculation of beta diversity before the results were visualized with principal coordinate analysis (PCoA). Additionally, the linear discriminant analysis effect size (LEfSe, LDA > 3) enabled the identification of important bacteria in the two groups, with the microbiome function subsequently predicted with PICRUSt 2 software. Significantly enriched pathways were eventually identified based on KEGG analyses.

### 2.6. Statistical Analysis

Excel 2010 was utilized for the preliminary sorting of the experimental data before it underwent statistically analysis with SPSS 26.0 (IBM, Armonk, NY, USA). All results are given as mean ± standard deviation, and, after using the Shapiro–Wilk and Levene tests to assess the normality and homogeneity of data variance, respectively, the results were analyzed using independent samples *t*-tests, with *p* < 0.05 representing significance.

## 3. Results

### 3.1. Growth Performance

As can be seen from Table 2, there was no significant difference in the body weight (BW) between the two experimental groups at 0, 30, 60, and 90 days (*p* > 0.05), but the BW increased faster in the HC group. Compared with the LC group, there was no significant difference in the ADFI for day 31–60 and 61–90 in the HC group (*p* > 0.05), but the ADFI for day 1–30 and 1–90 in the HC group was significantly increased (*p* < 0.05). For day 31–60 and 61–90, there was no significant difference in ADG between the two groups (*p* > 0.05), but for day 1–30 and day 1–90, the ADG in HC group was significantly increased (*p* < 0.05).

### 3.2. Serum Biochemical Indexes

It can be seen from Table 3 that the ALB concentration in the HC group was significantly higher than that in the LC group on day 60 and 90 of the experiment (*p* < 0.05). There were no significant differences in the concentrations of TP, GLB, CREA, ALT, and LDH between the LC group and the HC group on day 30, 60, and 90 (*p* > 0.05). At 30 d, 60 d, and 90 d, the concentrations of GLU and ALP in the HC group were higher than those in the LC group, but the differences were not significant (*p* > 0.05). The AST levels were consistently lower in HC, but the difference did not reach significance (*p* > 0.05).

### 3.3. Richness and Diversity of Rumen Bacteria

**Figure 1:**
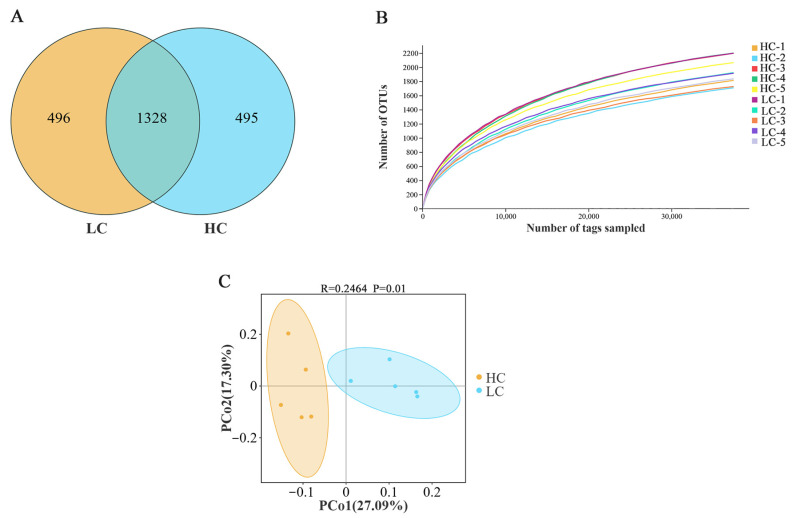
Microbial communities between the two groups. (**A**) Venn diagram illustrating unique and common OTUs between the groups. (**B**) The rarefaction curve for each sample in both groups. (**C**) PCoA using the weighted uniFrac distance. LC, low-dietary-concentrate-level group; HC, high-dietary-concentrate-level group.

The sequencing of the 16S rDNA in 10 samples of yak ruminal fluid yielded 1,018,274 sequences, of which 505,259 sequences were obtained in the LC group and 513,015 sequences were obtained in the HC group, with an average sequence length of 454.44 bp. As shown in Figure 1A, there were 2319 OTUs in the two groups of samples, among which 1328 OTUs, accounting for 57.27% of the total, were shared. The number of OTUs unique to the LC group was 496, and the number of OTUs unique to the HC group was 495. Rarefaction curves were constructed according to OTU numbers for the evaluation of sequencing depth. Figure 1B shows that increased sample numbers led to plateauing in the OTU numbers, suggesting sufficient sequencing depth. Table 4 shows the alpha diversity results. The HC group had a higher but non-significant Shannon index, Chao1 index, and ACE index compared with LC (*p* > 0.05). Beta diversity was analyzed using PCoA analysis based on a weighted Unifrac distance matrix. The PCoA results (Figure 1C) show that PCo1 and PCo2 contributed 27.09% and 17.30%, respectively, indicative of significant differences in the microbial compositions.

### 3.4. Structural Composition of Rumen Bacteria

**Figure 2:**
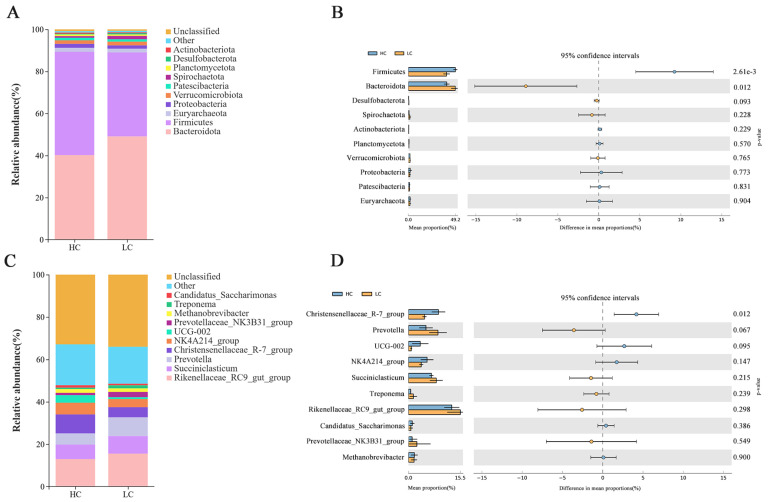
Ruminal bacterial compositions in the two groups. (**A**) Relative abundance of phyla. (**B**) Dominant phyla in the LC and HC groups. (**C**) Relative abundance of genera. (**D**) Dominant genera in the LC and HC groups.

Overall, this study identified 22 bacterial phyla in the HC and LC samples by means of identification analysis. Among them, the *Bacteroidota*, *Firmicutes*, *Euryarchaeota*, *Proteobacteria*, and *Verrucomicrobiota* phyla predominated. The community compositions of the ruminal bacteria, as well as the top 10 phyla and genera showing differential abundance, are illustrated in Figure 2. In all samples, *Bacteroidota* and *Firmicutes* dominated. *Bacteroidota* accounted for 49.44% and 40.53% of the identified phyla abundances in LC and HC, respectively, and *Firmicutes* accounted for 40.26% and 49.64% of the identified phylum abundances in LC and HC, respectively (Figure 2A). As seen in Figure 2B, *Firmicutes* were more abundant in HC relative to LC (*p* < 0.05), while *Bacteroidota* were more prevalent in LC (*p* < 0.05). Overall, 276 bacterial genera were identified. Both groups showed a predominance of *Rikenellaceae_RC9_gut_group* (24.03%, 19.68%), *Succiniclasticum* (12.90%, 10.44%), *Prevotella* (13.68%, 7.98%), *Christensenellaceae_R-7_group* (7.43%, 13.64%), and *NK4A214_group* (5.94%, 8.46%) in the ruminal contents (Figure 2C). *Rikenellaceae_RC9_gut_group*, *Succiniclasticum*, and *Prevotella* showed greater abundance in LC relative to HC (Figure 2D), although the change was non-significant (*p* > 0.05). Compared with LC, samples from HC showed higher proportions of *Christensenellaceae_R-7_group* (*p* < 0.05), and NK4A214_group was also higher, although the level did not reach significance (*p* > 0.05).

### 3.5. LEfse Analysis of Rumen Bacteria

**Figure 3:**
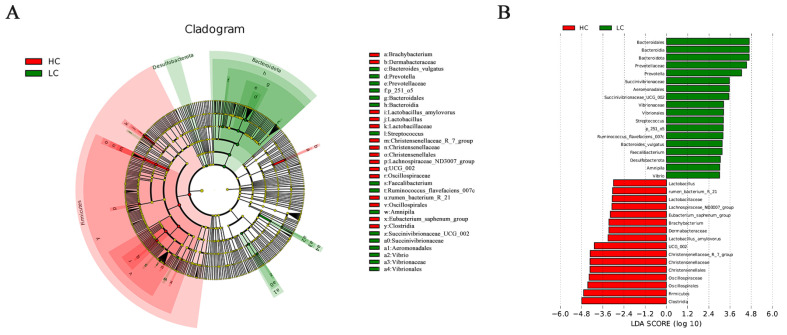
LEfSe analysis of ruminal bacteria in the LC and HC groups. (**A**) Evolutionary plots showing that the LC and HC groups were significantly different in terms of microbial species. (**B**) Linear discriminant analysis score histograms based on categorical information.

To better understand the specific major bacterial groups in the HC and LC groups, the LEfse method was used to characterize the bacteria from phylum to genus levels. Figure 3A illustrates the structures of the ruminal microbiota. The HC group had 15 more abundant branches, while 16 more branches were abundant in the LC group. The distribution results for the LDA values of different bacterial groups are presented in Figure 3B. Bacterial communities such as *Streptococcus*, *Prevotellaceae*, *Bacteroidia*, *Bacteroidota*, and *Prevotella* increased significantly in relative abundance in LC (LDA > 3, *p* < 0.05). In HC, bacterial communities such as *Christensenellales*, *Firmicutes*, *Christensenellaceae*, and *Christensenellaceae_r_7_group* increased significantly in their relative abundance (LDA > 3, *p* < 0.05).

### 3.6. PICRUSt 2 Function Prediction

**Figure 4:**
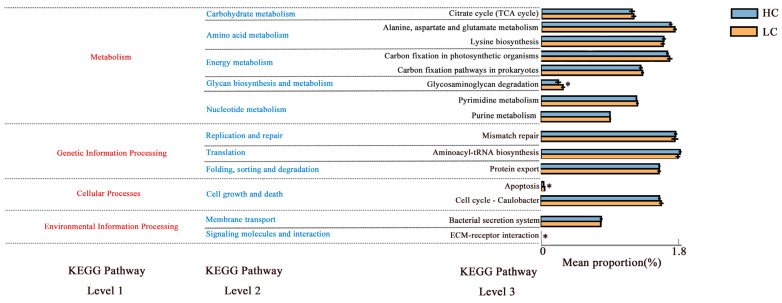
Prediction of ruminal bacterial function in the LC and HC groups, created by PICRUSt 2. * difference between the two groups is significant (*p* < 0.05).

To better understand the functions of the ruminal microflora, PICRUSt 2 was utilized for functional prediction using KEGG. As shown in Figure 4, the bacterial genes in the ruminal fluids of the two groups of yaks encompassed the four major biological metabolic pathways in Level 1 KEGG analysis, namely, metabolism, genetic information processing, cellular processes, and environmental information processing. Their average proportions were 79.28%, 14.30%, 4.04%, and 1.73%, respectively. KEGG Level 2 analysis showed that carbohydrate metabolism, amino acid metabolism, and energy metabolism were relatively abundant metabolic pathways in rumen microorganisms. At KEGG Level 3, the average abundance of glycosaminoglycan degradation, apoptosis, and ECM–receptor interaction in LC was significantly higher than that in HC (*p* < 0.05).

## 4. Discussion

The yaks in the HC group were found to have significantly higher ADFI and ADG values relative to those in the LC group throughout the experimental period, demonstrating that the increase in concentrate proportion in diets had positive effects on the DMI and ADG of yaks, which was consistent with the results of Chen et al. [7]. This is likely due to greater amounts of non-structural carbohydrate in high-concentrate diets, as its degradation rate is faster than that of structural carbohydrate, which improves the nutrient intake and growth performance of yaks [27]. At the same time, we also found that the ADG of yaks in the LC group and the HC group gradually decreased with the progress of the experiment, which may be due to the sharp drop in environmental temperature in the later stage of the experiment, as yaks needed to spend a lot of energy on maintaining body temperature, resulting in weight reduction in the two groups. In conclusion, suitably raised amounts of concentrate in the diet enhance the palatability of the food, increase the rate of chyme flow, and improve digestibility, thereby improving growth performance [7].

Blood metabolites are important indicators of animal nutrition and physiological status, which can reflect the health status of some tissues and organs in animals [7,28]. Serum total protein is composed of albumin and globulin, which maintain the normal movement pressure and pH value of intravascular colloid, transport a variety of metabolites, and reflect protein digestion and absorption in animals [11,29]. At 60 d and 90 d, the HC group had higher concentrations of TP and ALB compared with the LC group, possibly due to enhanced protein synthesis and humoral immunity in the animas after adding the 60% concentrate [30,31,32]. Blood glucose concentration can reflect the energy utilization of ruminants, with reduced concentrations suggesting the poor energy utilization of inadequate dietary energy [6,33]. As expected, the HC group had elevated GLU levels relative to the LC group at all three experimental stages, indicating that increasing the concentrate level can increase glucose concentration in the serum of yaks, thus providing more energy for the body. The activities of ALT and AST, which are released into the blood following liver damage, can reliably reflect the status of liver functions [34]. ALT and AST increased, indicating different degrees of liver damage. Alkaline phosphatase is widely distributed in liver, bone, and other tissues and is excreted from the bladder through the liver; it represents an indicator of liver functioning and protein metabolism [5,11]. It was found that the ALT, AST, and ALP levels in the groups were comparable, with both showing normal levels and normal liver function. LDH levels indicate inflammation and cell damage in clinical diagnoses [5]. Using buffalo LDH levels between 186.72 and 917.43 (U/L) [35] as a reference, it was found that the serum LDH levels in both groups exceeded the normal range on day 30 and 60 of the experiment, which may be due to cellular inflammatory responses, leading to increased LDH levels [5].

The rumen of ruminants contains high-density microbiomes, including bacteria, protozoa, archaea, and fungi [36,37]. There is a mutualistic symbiotic relationship between ruminants and rumen microorganisms [38]. Ruminants provide nutrients and a stable ruminal environment for microbial growth and reproduction [39]. Ruminal microorganisms degrade food and produce hydrogen, lipid, amino acids, lactic acid, and volatile fatty acids, which provide energy and enhance the production performance of ruminants [40]. Studies have shown that dietary nutrition levels largely influence the health status, rumen microbial diversity, and productivity of ruminants [41,42]. In high-throughput sequencing, the Shannon index reflects species diversity, and the ACE index and Chao1 index reflect species richness. Pang et al. [10] showed that a high-concentrate group exhibited significantly lower alpha diversity indexes (Shannon and Chao1) compared with a low-concentrate group. In contrast, this study found the HC group to have higher but non-significant Shannon, Chao1, and ACE indexes in comparison with the LC group, suggesting that appropriate increases in the amount of concentrate in the diet could enhance the diversity and richness of the ruminal bacteria in the yak. This apparent inconsistency may have resulted from differences in the diets. Although the HC and LC groups did not differ significantly in terms of microbial richness and diversity, there were significant effects on Beta diversity and community composition differences, with PCoA analysis revealing two clearly separated groups.

Here, *Bacteroidota* and *Firmicutes* predominated in both groups, with similar results reported by Dai [6] and Yi et al. [8]. *Firmicutes* and *Bacteroidota* are known to degrade fibrous substances and non-fibrous carbohydrates, respectively, with both bacterial phyla being also involved in the nutrient metabolism of ruminants [43]. As described by Pang et al. [10], this study also noted a significant reduction in the proportions of *Bacteroidota*, while *Firmicutes* were increased in HC. However, Mao et al. observed a significantly lower abundance of *Firmicutes* but increased proportions of *Bacteroidota* in the rumina of the high-concentrate group [18]. This inconsistency could be attributed to the fact that most *Bacteroidota* bacteria are Gram-negative bacteria. The lower ruminal pH value of the HC group leads the degradation of Gram-negative bacteria in large numbers and finally leads to a decline in the relative abundance of *Bacteroidota*, while the relative abundance of *Firmicutes* increases accordingly [44].

Similarly to the findings of Pang [10] and Liu et al. [5], the dominant bacterial genera in yak rumen are *Rikenellaceae_RC9_gut_group*, *Succeniclasticum*, *Prevotella*, *Christensenellaceae_R-7_group*, and *NK4A214_group*. According to previous reports, the *Rikenellaceae_RC9_gut_group*, which is assigned to *Bacteroidota*, can degrade polysaccharides, with a higher dietary fiber content often increasing its relative abundance [2,6]. It was found that *Rikenellaceae_RC9_gut_group* was more abundant in LC rather than HC rumina, probably due to the higher crude fiber content in the LC group. It is reported that *Succiniclasticum* can produce succinate in ruminants and convert it to propionate and then produce glucose [45]. It is found that the rumen *Succiniclasticum* content increased at greater concentrate levels [6], differing from the present results. This could be attributed to the different physiological stages, dietary structures, and ages of the research animals.

*Prevotella* is known to be involved in degrading and using nutrients such as xylans, starches, proteins, and non-cellulosic polysaccharides [41,46]. The increase with higher ratios of concentrate raised the proportion of *Prevotella*, probably due to higher starch and lower fiber content [18]. However, here, HC had a lower proportion of *Prevotella* relative to LC, as previously reported by Chen et al. [46]. The reason may be that the low rumen pH caused by a high-concentrate diet affects its abundance. It is reported that *Christensenellaceae_R-7_group* can promote rumen development and enhance nutrient digestion and absorption in ruminants, with protein catabolism and intestinal metabolism in feed positively influencing its relative abundance. HC also had a significantly greater proportion of *Christensenellaceae_R-7_group* compared with LC, indicating that feeding a certain degree of high-concentrate diet can enhance protein metabolism in yaks. It was previously reported that the amount of dietary concentrate raised the proportion of *NK4A214_group* [8], with similar findings obtained in the current work. *NK4A214_group* can degrade or utilize degradation-resistant polysaccharides and fibers in feed [47]. The difference in the abundance of *NK4A214_group* is related to the higher amount of resistant starch in the high-concentrate diet.

The analysis of predictive functions revealed that level 1 genes were largely consistent across the two groups of diets, while the proportion of concentrate in the diet directly affected the level 2 and level 3 gene function profiles of rumen microbiome in yaks. The LC group showed significant enrichment in ECM–receptor interactions relative to HC. Through the comparative transcriptome analysis of adipose tissue, Lee et al. [48] found that, in cattle, ECM–receptor interactions played a key role in depot-specific adipogenesis. The average abundance of ECM–receptor interactions in LC was significantly increased, suggesting that the ECM–receptor interaction pathway could be a key one that regulates meat quality in yaks. However, our results are only based on the predicted 16S rDNA and may not reflect the actual function of rumen bacteria. Hence, further metagenomic analysis is required to evaluate the associations between concentration and these genes.

At present, although there are many experiments on the effects of different proportions of concentrate on various indexes in ruminants, most of them focus on the analysis of one or several performance of ruminants, and there is not sufficient research on the mechanism. At the same time, the production performance of different ruminants varies in response to varying proportions of concentrate. Therefore, subsequent studies can combine multi-omics analysis and other methods to further reveal how dietary structures influence animals and provide theoretical support for the mechanisms underlying the influence of the concentrate-to-forage ratio in the diet on the growth and ruminal microflora of ruminants.

## 5. Conclusions

Diets containing a high level of concentrate (concentrate–forage = 60:40) significantly increased the ADFI and ADG and improved serum biochemical indices and the growth performance of house-fed yaks. The community structure of rumen flora was also altered after feeding with the high-concentrate diet, with an increase in the relative abundance of *Firmicutes*, the *NK4A214_group*, and the *Christensenellaceae_R-7_group*, as well as a reduced abundance of *Bacteroidota*, *Prevotella*, *Succiniclasticum*, and the *Rikenellaceae_RC9_gut_group*. These changes in microflora further affected the function of the yak rumen microbiota.

## Figures and Tables

**Table 1:** Composition and nutritional levels of the diets (DM%).

Items	Group ^2^	
	LC	HC
	Diet composition	
Alfalfa hay	30.00	20.00
Corn straw	30.00	20.00
Concentrate ^1^	40.00	60.00
Total	100.00	100.00
	Nutrient levels	
Dry matter	91.30	90.20
Crude protein	13.08	13.02
Ether extract	1.70	2.10
Neutral detergent fiber	45.20	37.10
Acid detergent fiber	26.90	19.30
Calcium	0.96	0.84
Phosphorus	0.34	0.38
ME, MJ/kg ^3^	9.45	9.50
Concentrate–forage	40:60	60:40

^1^ The concentrate consisted of corn, soybean meal, cottonseed meal, DDGS, bran, molasses, corn husk, soybean oil, extruded urea, fine stone powder, calcium hydrogen phosphate, sodium bicarbonate, magnesium oxide, sodium chloride, and premix. The premix composition, per kg of diet, was as follows: Co 56 mg; I 193 mg; Fe 5600 mg; Se 35 mg; Zn 9100 mg; Cu 2310 mg; Mn 4900 mg; VA 600,000 IU; VD 3,240,000 IU; VE 1800 IU; D-biotin 2 mg; and Nicotinamide 1800 mg. ^2^ LC, low-dietary-concentrate-level group; HC, high-dietary-concentrate-level group. ^3^ ME, metabolizable energy.

**Table 2:** Effect of a high-concentrate diet on yak growth performance.

Items ^1^	Experimental Stage	Group ^2^		*p*-Value
		LC	HC	
BW (kg)	0 d	151.86 ± 14.73	151.61 ± 15.43	0.973
	30 d	173.98 ± 14.85	186.32 ± 14.53	0.115
	60 d	195.63 ± 19.52	205.54 ± 14.72	0.270
	90 d	202.59 ± 17.83	216.34 ± 15.73	0.124
ADFI (kg/d)	1–30 d	5.24 ± 0.77	5.65 ± 0.75 *	0.004
	31–60 d	5.96 ± 0.56	6.08 ± 0.54	0.238
	61–90 d	5.76 ± 0.60	5.70 ± 0.61	0.631
	1–90 d	5.62 ± 0.75	5.84 ± 0.64 *	0.005
ADG (kg/d)	1–30 d	0.74 ± 0.25	1.22 ± 0.07 *	<0.001
	31–60 d	0.72 ± 0.23	0.64 ± 0.19	0.459
	61–90 d	0.23 ± 0.19	0.36 ± 0.22	0.230
	1–90 d	0.56 ± 0.13	0.72 ± 0.12 *	0.026

^1^ BW, body weight; ADFI, average daily feed intake; ADG, average daily gain. ^2^ LC, low-dietary-concentrate-level group; HC, high-dietary-concentrate-level group; * means that the difference between the two groups is significant (*p* < 0.05).

**Table 3:** Effect of a high-concentrate diet on the serum biochemical indices of yak.

Items ^1^	Experimental Stage	Group ^2^		*p*-Value
		LC	HC	
TP (g/L)	30 d	69.01 ± 7.58	64.96 ± 4.61	0.218
	60 d	64.64 ± 4.16	67.00 ± 4.20	0.279
	90 d	61.92 ± 5.90	66.69 ± 5.18	0.108
ALB (g/L)	30 d	33.14 ± 4.19	32.76 ± 2.24	0.822
	60 d	32.05 ± 1.95	35.31 ± 2.98 *	0.021
	90 d	31.19 ± 2.90	35.25 ± 0.38 *	0.009
GLB (g/L)	30 d	35.86 ± 6.95	32.20 ± 3.68	0.209
	60 d	32.59 ± 4.77	31.69 ± 4.51	0.702
	90 d	30.72 ± 5.06	31.44 ± 4.50	0.768
CREA (umol/L)	30 d	97.66 ± 15.84	93.73 ± 6.43	0.551
	60 d	101.29 ± 9.99	112.60 ± 20.62	0.184
	90 d	106.90 ± 15.27	117.16 ± 18.10	0.241
GLU (mmol/L)	30 d	4.95 ± 0.36	5.34 ± 0.45	0.070
	60 d	4.11 ± 0.27	4.20 ± 0.31	0.572
	90 d	4.17 ± 0.47	4.20 ± 0.30	0.894
AST (U/L)	30 d	100.74 ± 22.18	96.77 ± 15.05	0.681
	60 d	99.06 ± 22.03	98.63 ± 14.43	0.964
	90 d	87.27 ± 18.06	82.07 ± 13.45	0.543
ALT (U/L)	30 d	34.67 ± 14.39	36.33 ± 0.79	0.804
	60 d	36.35 ± 3.01	31.11 ± 10.32	0.219
	90 d	29.21 ± 10.17	30.95 ± 7.86	0.708
ALP (U/L)	30 d	161.19 ± 28.67	176.74 ± 16.79	0.231
	60 d	153.32 ± 34.32	182.16 ± 67.64	0.300
	90 d	131.24 ± 27.41	157.73 ± 66.89	0.318
LDH (U/L)	30 d	1017.40 ± 135.52	970.06 ± 151.37	0.520
	60 d	953.50 ± 141.00	1022.33 ± 167.00	0.388
	90 d	818.85 ± 82.20	874.96 ± 100.02	0.261

^1^ TP, total protein; ALB, albumin; GLB, globulin; CREA, creatinine; GLU, glucose; AST, aspartate aminotransferase; ALT, alanine aminotransferase; ALP, alkaline phosphatase; LDH, lactate dehydrogenase. ^2^ LC, low-dietary-concentrate-level group; HC, high-dietary-concentrate-level group; * means that the difference between the two groups is significant (*p* < 0.05).

**Table 4:** Alpha diversity indices of ruminal bacteria from the two groups.

Items	Group ^1^		*p*-Value
	LC	HC	
Shannon	7.76 ± 0.39	7.96 ± 0.39	0.492
Chao1	2332.36 ± 139.11	2397.05 ± 128.47	0.514
ACE	2455.60 ± 144.67	2538.95 ± 145.74	0.440

^1^ LC, low-dietary-concentrate-level group; HC, high-dietary-concentrate-level group.

## Data Availability

The data that support the findings of this study are available from the corresponding author upon reasonable request. The sequencing data are available in the NCBI under BioProject number PRJNA1173922.
